# Mental Health Impacts of Quarantine: Insights from the COVID-19 International Border Surveillance Study in Toronto, Canada

**DOI:** 10.1192/j.eurpsy.2022.368

**Published:** 2022-09-01

**Authors:** C. Regehr, V. Goel, E. De Prophetis, M. Jamil, D. Mertz, L. Rosella, D. Bulir, M. Smieja

**Affiliations:** 1 University of Toronto, Factor-inwentash Faculty Of Social Work, Toronto, Canada; 2 University of Toronto, Dalla Lana School Of Public Health, Toronto, Canada; 3 McMaster University, Mcmaster Health Labs, Hamilton, Canada; 4 McMaster University, Department Of Medicine, Hamilton, Canada; 5 McMaster University, Research Institute Of St Joes, Hamilton, Canada; 6 McMaster University, Pathology And Molecular Medicine, Hamilton, Canada

**Keywords:** covid, quarantine, mental health

## Abstract

**Introduction:**

Nations thorughout the world are imposing mandatory quarantine on those entering the country. While such measures may be effective in reducing the importation of COVID-19, the mental health implications remain unclear.

**Objectives:**

This study sought to assess mental well-being and factors associated with changes in mental health in individuals subject to mandatory quarantine following travel.

**Methods:**

Travellers arriving at a large urban international airport completed online questionnaires on arrival and days 7 and 14 of mandated quarantine. Questionnaire items such as travel history, mental health, attitudes towards COVID-19, and protection behaviours were drawn from the World Health Organization Survey Tool for COVID-19.

**Results:**

There was a clinically significant decline in mental health over the course of quarantine among the 10,965 eligible participants. Poor mental health was reported by 5.1% of participants on arrival and 26% on day 7 of quarantine. Factors associated with greater decline in mental health were younger age, female gender, negative views towards quarantine measures, and engaging in fewer COVID-19 prevention behaviours.

**Conclusions:**

While the widespread use of quarantine may be effective in limiting the spread of COVID-19, the mental health implications are profound and have largely been ignored in public policy decision-making. Psychiatry has a role to play in contributing to the public policy debate to ensure that all aspects of health and well-being are reflected in decisions to isolate people from others.

**Disclosure:**

No significant relationships.

